# Supplementary material for: Topologically protected modes in non-equilibrium stochastic systems
Source: Nat Commun. 2017 Jan 10;8:13881. doi: 10.1038/ncomms13881 (PMC5234070; doi:10.1038/ncomms13881)
Supplement: Supplementary Information — Supplementary Figures 1-6, Supplementary Notes 1-5, Supplementary Discussion and Supplementary Reference [file ncomms13881-s1.pdf]

# Supplementary Information

Arvind Murugan and Suriyanarayanan Vaikuntanathan*
*The University of Chicago, Chicago, IL, 60637*

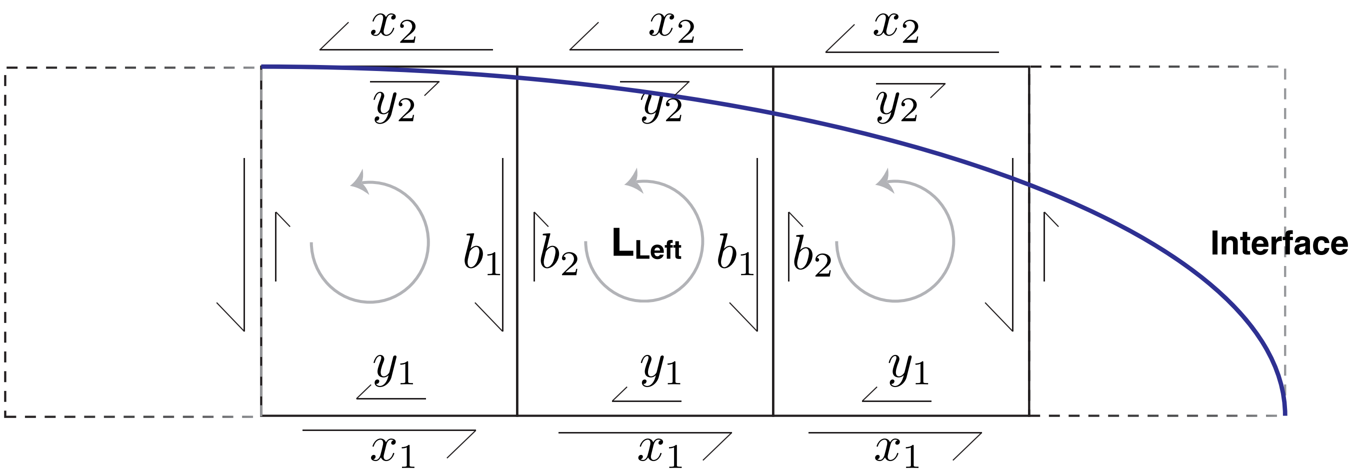


SUPPLEMENTARY FIG 1. Toy network introduced in the main text with one boundary. In this case, the right network $W_R$ is simply vacuum. We are interested in conditions under this network can support a localized edge mode at the right edge (blue highlighted curve).

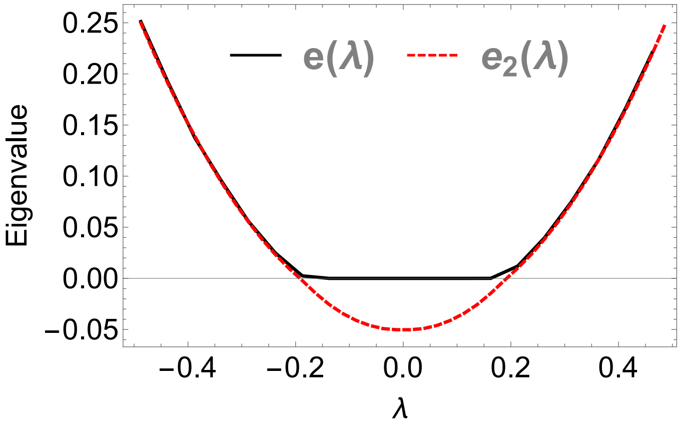


SUPPLEMENTARY FIG 2. The first two eigenvalues of $W(\lambda)$. We have plotted the real part of the second eigenvalue. The parameters are the same as those in Fig 2. As in the main text, we simulated a system in which two bulk networks $L$ and $R$ are connected by interfaces. The number of vertical rungs in the network is $N = 200$.

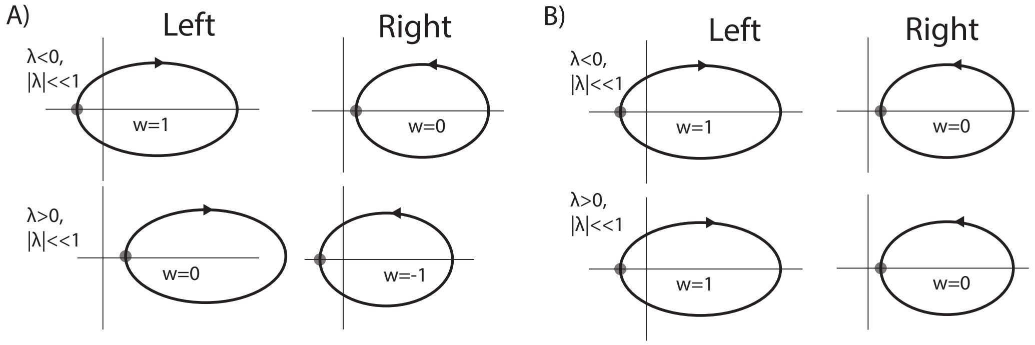


SUPPLEMENTARY FIG 3. A sketch of configurations that can result in a winding number mismatch of $\delta w = 1$ and have a non-zero $\lambda^+$ and $\lambda^-$. The x-axis in these plots denotes the real part of the determinant of $W_{L/R}(\lambda, k)$, and the y axis denote the imaginary part. The dot on the axis marks the value of the cumulant generating function for $|\lambda| \ll 1$. In case (A) the cumulant generating function for both the bulk regions changes sign as $\lambda$ crosses zero and hence has a non-zero slope. In case (B), the cumulant generating function doesn't change sign as $\lambda$ crosses zero. Such bulk networks support zero current in their steady state. However, the sketches in B) are not allowed since the second derivative of the cumulant generating function is constrained to be non-negative [1]. One of the configurations in B) violates this constraint. Hence, the slope of the cumulant generating function for the bulk networks has to be non-zero in order for the combined network to have a non-zero $\lambda^+$ and $\lambda^-$.

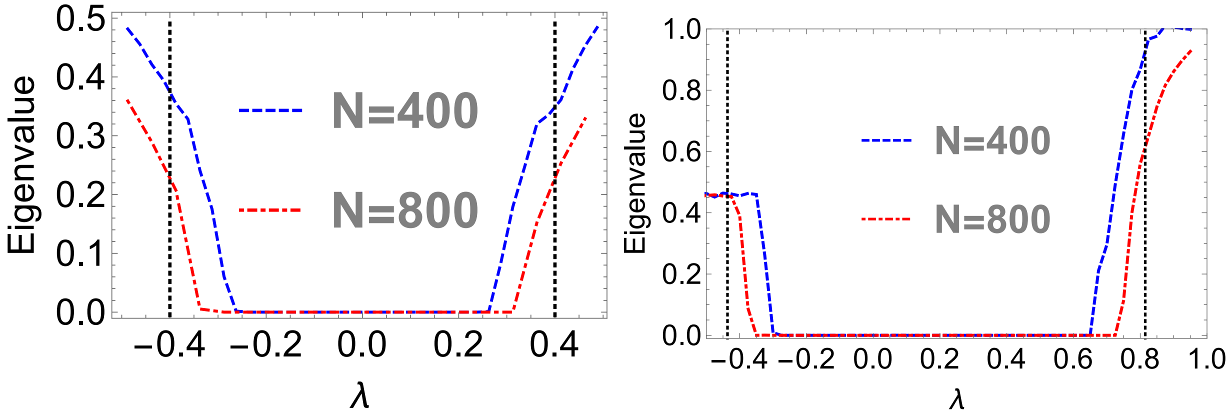


SUPPLEMENTARY FIG 4. (Real part of) Lowest eigenvalue by magnitude of $W(\lambda)$ for two different networks. The value of $\lambda_\pm$ for these parameters is marked by the black dashed vertical lines in the figure. As in the main text, the theoretical prediction for $\lambda_\pm$ is approached with increasing system size. As in the main text, we simulated a system in which two bulk networks $L$ and $R$ are linked by interfaces.

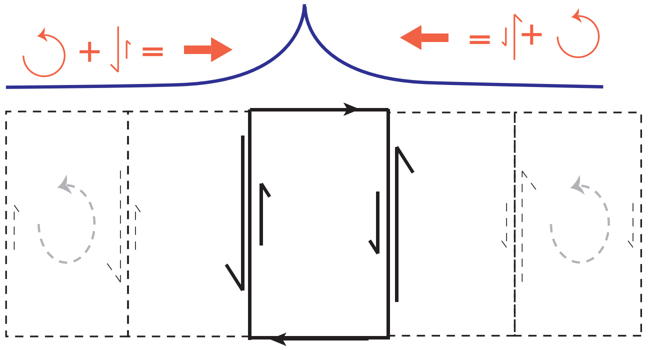

SUPPLEMENTARY FIG 5. When the winding number mismatch is $\delta w = 1$, the probability distribution is localized and the properties of the adaptive network can be studied in terms of a smaller network. In the limit of strong localization, we can simply consider a four state network as highlighted above.

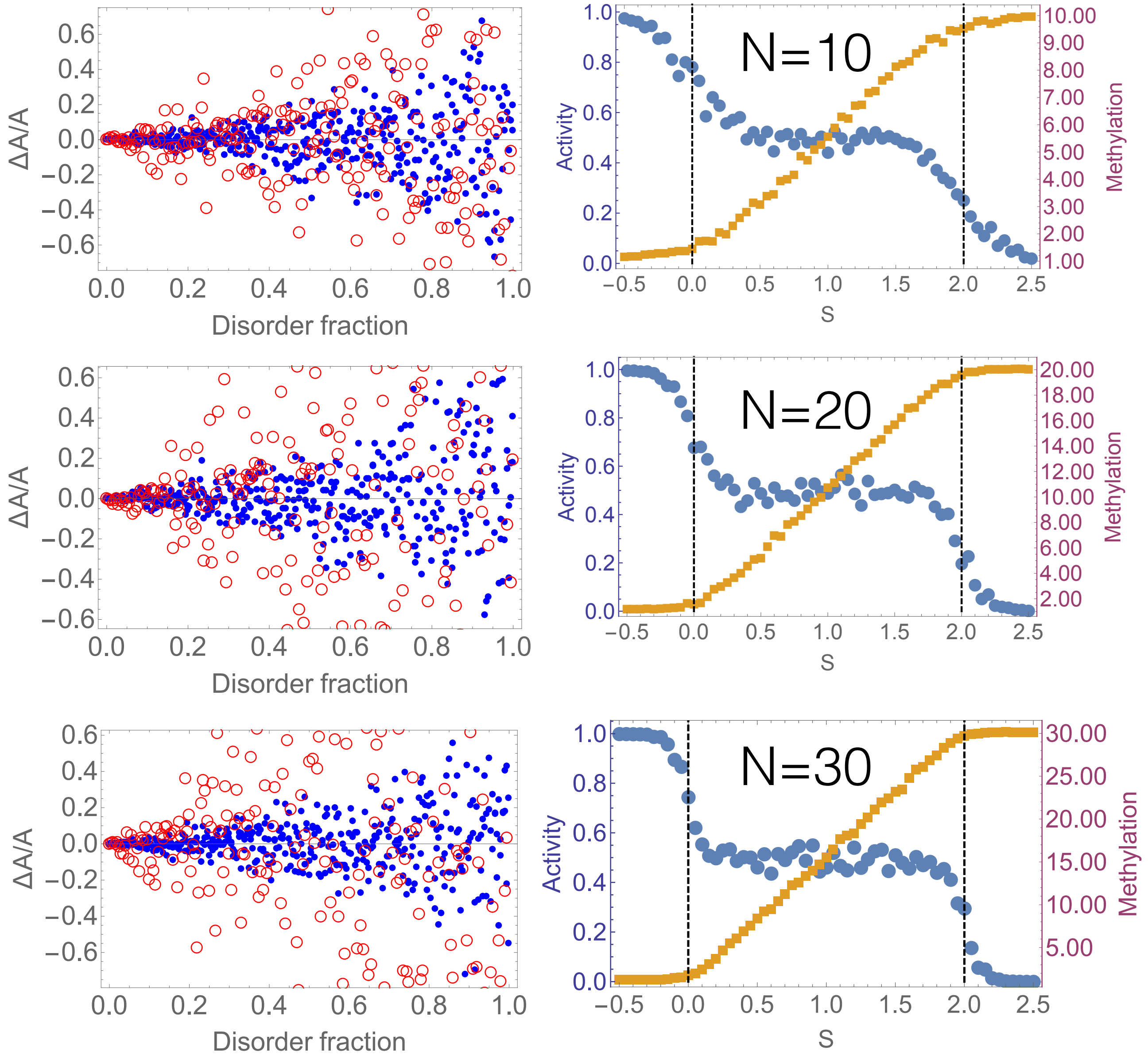


SUPPLEMENTARY FIG 6. Robust adaptation in finite networks. Topologically protected modes are present even at $N = 10$ as evidenced by the insensitivity of the network output to quenched disorder in the rates.

## SUPPLEMENTARY NOTE 1: BULK BOUNDARY CORRESPONDENCE FOR AN INTERFACE BETWEEN NETWORK AND VACUUM

We derive conditions under which the presence of the boundary mode is predicted by the eigenvalue spectrum in the bulk of the network. We work with an analogue of the adaptation network presented in the main text but with one boundary. In this case, the right network $W_R$ is simply vacuum. Networks with such topologies are present in models of kinetic proofreading [2].

For such ladder networks we consider the cumulant generating function associated with current fluctuations along the $x$ axis. This cumulant generating function is simply the largest eigenvalue of the tilted transition matrix, $W(\lambda)$, with elements

$$W(\lambda)_{i,j} = W_{i,j} e^{\lambda(x_i - x_j)} \tag{1}$$

where $x_i$ denotes the location of the node $i$ along the x-axis, and $W_{i,j}$ denotes the rate of transition from node $j$ to node $i$. The transition rates have been specified in Supplementary Fig. 1.

We now show number of localized edge modes of the matrix $W(\lambda)$ can be related to a *bulk* topological quantity. In other words, the character of the modes of the finite matrix $W(\lambda)$ —which is composed of a periodically replicated *bulk* connected to *vacuum* by an interface —can be inferred by studying the spectrum of the translationally invariant *bulk* phase. This result establishes the topological character for edge modes in non-equilibrium systems.

Bulk topological phases, by definition, have translational invariance. Hence $W(\lambda)$ for a pure bulk phase can hence be studied in momentum space:

$$W(\lambda, k) = \begin{pmatrix} -b_1 + \left(-1 + e^{\lambda - ik}\right) x_1 + \left(-1 + e^{ik-\lambda}\right) y_1 & b_2 \\ b_1 & -b_2 + \left(-1 + e^{ik-\lambda}\right) x_2 + \left(-1 + e^{\lambda - ik}\right) y_2 \end{pmatrix} \tag{2}$$

$$\equiv \begin{pmatrix} -b_1 + \left(-1 + ze^{\lambda}\right) x_1 + \left(-1 + \frac{e^{-\lambda}}{z}\right) y_1 & b_2 \\ b_1 & -b_2 + \left(-1 + \frac{e^{-\lambda}}{z}\right) x_2 + \left(-1 + e^{\lambda} z\right) y_2 \end{pmatrix}. \tag{3}$$

where we have introduced $z = \exp(-ik)$ and note that $W(\lambda, k) \equiv W(\lambda, z)$ is an analytic operator in $z$.

Note that $W(\lambda, z)$ is a $2 \times 2$ matrix in momentum space because the ladder has two states in the transverse direction. For example, the eigenvalue problem in momentum space can be re-expressed as

$$W(\lambda, z) \begin{pmatrix} u \\ 1 \end{pmatrix} = \phi(\lambda, z) \begin{pmatrix} u \\ 1 \end{pmatrix} \tag{4}$$

where $\binom{u}{1}$ represents the relative distribution between the two horizontal rails of the ladder while the eigenvalue $\phi(\lambda, z)$ encodes the spatial structure of eigenvectors along the ladder (horizontal in Supplementary Fig. 1). **In particular, zeromodes of $W(\lambda)$ that decay as $e^{-\gamma x}$ in real space, with $\gamma > 0$, show up as zeros of** $\det W(\lambda, z)$ **contained inside the unit circle** $|z| = 1$. Hence we can count the number of such zeros using Cauchy's residue theorem,

$$\delta w = \frac{1}{2\pi i} \int \frac{d}{dz} \ln \det[W(\lambda, z)] = \frac{1}{2\pi i} \int_0^{2\pi} \mathrm{Tr}[W(\lambda, k)^{-1} \partial_k W(\lambda, k)] \tag{5}$$

where we choose an integration contour along the unit circle $|z| = 1$ .

The function $\det W(\lambda, z)$ has two poles at the origin. Hence, if the $\det[W(\lambda, e^{ik})]$ rotates around the origin counter-clockwise once as $k$ is varied from $k = 0$ to $k = 2\pi$, then $\delta w = 1$ and $\phi(\lambda, z)$ has three zeros within the unit circle. If $\det[W(\lambda, e^{ik})]$ rotates around the origin clockwise once as $k$ is varied from $k = 0$ to $k = 2\pi$, then $\delta w = -1$ and $\phi(\lambda, z)$ has one zero within the unit circle. $\phi(\lambda, z)$ has two zeros in the case that $\det[W(\lambda, e^{ik})]$ doesn't wind around the origin. We now consider the constraints imposed on the spectrum of $W(\lambda)$ when 1) $\delta w = 1$, 2) $\delta w = -1$ and 3) $\delta w = 0$.

1. $\phi(\lambda, z)$ has three localized zeros if $\delta w = 1$. In this case a linear combination of these zero modes is sufficient to construct a localized zero eigenmode for any finite matrix $W(\lambda)$. This implies that $W(\lambda)$ **has a localized mode at its edge whenever** $\delta w = 1$.

2. $\phi(\lambda, z)$ has one localized zeros if $\delta w = -1$. This solution alone is not sufficient to construct a localized edge mode of the finite matrix $W(\lambda)$. Hence $W(\lambda)$ **has no localized edge modes when** $\delta w = -1$.

3. $\phi(\lambda, z)$ has two localized zeros if $\delta w = 0$. A linear combination of two zero modes is not sufficient to construct a localized mode for any arbitrary choice of boundaries in the finite matrix $W(\lambda, z)$. The character of the edge modes in this marginal regime is sensitive to boundary conditions. Systems with $\delta w = 0$ **in the bulk do not have topologically protected edge modes.**

Eq. 5 is a special case of the index theorem derived in the main text for conditions in which one of the interfaces is vacuum. The arguments presented above clearly show that $\delta w = \mathrm{ind} W$. These arguments prove that the number of localized edge modes in a non-equilibrium network can be related to a topological quantity computed in the bulk of the network.

## SUPPLEMENTARY NOTE 2: BULK BOUNDARY CORRESPONDENCE FOR ARBITRARY BOUNDARIES

We now generalize these ideas to arbitrary boundaries between networks and not just a boundary with vacuum as discussed here. We begin by first constructing the Hermitian operator (as done earlier in [3] for mechanics),

$$H = \begin{pmatrix} 0 & W(\lambda) \\ W(\lambda)^T & 0 \end{pmatrix} \tag{6}$$

We are interested in zero eigen modes of $W(\lambda)$ confined to the interfacial region between two networks. As in the main text, we use the matrix diagonal matrix $\rho$ which has non zero elements $\rho_{i,i} = 1$ for nodes $i$ in the boundary region [3] to specify the interfacial region. In order to understand the topological nature of the zero modes of $W(\lambda)$, we compute its local index. This number is simply the difference between the number of zero eigenmodes of $W(\lambda)$, $n_+$, and the number of zero modes of $W(\lambda)^T$, $n_-$, in the region specified by $\rho$. This difference can be computed indirectly by computing the trace

$$n_+ - n_- = \lim_{i\epsilon \to 0} Tr\left[\rho \tau_z \frac{i\epsilon}{H + i\epsilon}\right] \tag{7}$$

where $\tau_z$ is the Pauli z matrix. We note that the operator $H$ satisfies the anti commutation relation $\{\tau_z, H\} = 0$ and possesses the so called particle hole symmetry. It was shown in [3] that the trace count for operators satisfying this symmetry can be rewritten as

$$n_+ - n_- = \frac{1}{2} \lim_{i\epsilon \to 0} Tr\left[\tau_z \frac{i\epsilon}{H + i\epsilon}\,[\rho, H]\right] \tag{8}$$

.

The elements of the commutator in the above equation, $[\rho, H]_{i,j}$ are simply $H_{i,j}(\rho_i - \rho_j)$. Approximating this as $H_{i,j}(\rho_i - \rho_j) \approx H_{i,j}(i-j)\nabla\rho$, we recognize that the trace count in Eq. 8 is non zero only in boundary region between the two bulks. Following the algebraic arguments presented in Ref [3], we can express this trace count in terms of the properties of the two bulk phases,

$$n_+ - n_- = w_L - w_R \tag{9}$$

where the *winding numbers* are defined as

$$w_{L/R} = \frac{1}{2\pi i}\int_0^{2\pi} dk \mathrm{Tr}[\mathrm{W_{L/R}(\lambda, k)^{-1}\partial_k W_{L/R}(\lambda, k)}]\,. \tag{10}$$

Here, $L$ and $R$ are meant to denote the two periodic networks that form $W$ and $W_{L/R}(k)$ denotes the Fourier transform of the respective matrix taken in the bulk region.

The difference of *winding numbers* $\delta w = w_L - w_R$ is a topologically protected object. If the winding number difference $\delta w = 1$ for an interval $\lambda_- < 0 < \lambda_+$ around the origin, then this analysis predicts that $W(\lambda)$ has atleast one topologically protected zero mode (Supplementary Fig. 4).

## SUPPLEMENTARY NOTE 3: ZEROS IN THE MAXIMAL EIGENVALUE OF $W(\lambda)$

In fact, the Perron Frobenius theorem constraints the number of protected modes to at most one and requires that the cumulant generating function for current fluctuations be zero in the interval $\lambda_- < 0 < \lambda_+$.

Our proof relies on the Perron Frobenius theorem which states that a positive definite matrix has a unique maximal real eigenvalue. While $W(\lambda)$ has negative elements along the diagonal, it can be shown [1] that $\exp(W(\lambda))$ is constrained to be positive. If the spectrum of eigenvalues is constructed as a function of $\lambda$, the Perron Frobenius theorem prohibits any eigenvalue crossing between the first (maximal) and second eigenvalues. Since the maximal eigenvalue of $W(0)$ is zero, the constraints imposed by the Perron Frobenius theorem require zero eigenvalue predicted by the winding number analysis for $\lambda_- < 0 < \lambda_+$ is necessarily the maximal eigenvalue. Hence we can prove that the winding number analysis identifies an interval $\lambda_- < 0 < \lambda_+$ for which the cumulant generating function is zero.

## SUPPLEMENTARY NOTE 4: LOCALIZATION OF THE STEADY STATE PROBABILITY

In this section, we argue that the steady state of the master equation in Eq 1 (main text) is localized if $W(\lambda)$ has localized zero modes for values of $\lambda$ around $\lambda = 0$, $\lambda^- < 0 < \lambda^+$.

As discussed in the main text, the existence of such an interval implies that the average current along the horizontal axis is zero in its steady state, $J = 0$. Given the translationally symmetry of the bulk, the allowed steady solutions in the bulk regions for Eq 1 (main text) are of the form $p_{\rm ss} = \{\ldots e^{\kappa i} \ldots\}$, where $i$ denotes the position of a node. The possibilities given this solution are a) $\kappa \neq 0$ , exponential decay and b) $\kappa = 0$, delocalized states.

We now rule out possibility b). We begin by considering a Markov state model composed only of one bulk translationally symmetric network. The steady state solution of such a Markov state model is necessarily characterized by $\kappa = 0$ and has translational symmetry. Further, if $\lambda^-, \lambda^+$ for the master equation in Fig 1 (main text) are non-zero, the corresponding bulk translationally symmetric networks must support a non-zero current in their translationally symmetric steady state. To see this, consider the cumulant generating function for the bulk networks. In Supplementary Fig. 3 we show that the existence of non-zero $\lambda^\pm$ requires that this bulk cumulant generating function has a non zero slope at $\lambda = 0$. Since the slope of the cumulant generating function is related to the average current in the steady state, the bulk networks support a non-zero current along the x-axis in their translationally symmetric state.

These arguments establish that the $\kappa = 0$ delocalized solution requires that the network in Fig 1 (main text) supports a non-zero current. Since our arguments establish that the steady current is $J = 0$, we can rule out the delocalized solution. The only other real steady state solutions allowed are localized exponential solutions, $\kappa \neq 0$.

## SUPPLEMENTARY NOTE 5: A HEURISTIC ESTIMATE FOR LOCALIZATION LENGTH

In the case of a one dimensional or pseudo one-dimensional networks, a simply heuristic connection between the points $\lambda^\pm$ and localization length can be derived. Specifically, in the case that network supports a localized mode with localization length $\eta$ in its steady state, the probability distribution is given by $p(x) \sim \exp(-|x|/\eta)$. We have assumed without loss of generality that localization is about the origin at $x = 0$. In this case, the cumulant generating function $e(\lambda) = -\lim_{\tau\to\infty} \ln\langle \exp(-\lambda j\tau)\rangle/\tau$ where $j\tau$ is the total displacement along the x axis in a time $\tau$ can be approximated as

$$e(\lambda) \sim -\lim_{\tau\to\infty} \frac{\ln\left[\int \exp(-\lambda j\tau)\exp(-|j\tau|/\eta) d(j\tau)\right]}{\tau} \tag{11}$$

This integral diverges when $\lambda^\pm > 1/\eta$ indicating that the exponential solution is no longer appropriate. Hence, the points $\lambda^\pm$ can be regarded as proxies for the localization length.

## SUPPLEMENTARY DISCUSSION 1: CONNECTION BETWEEN BAND GAPS AND TOPOLOGICALLY PROTECTED MODES

The results of the previous sections support the existence of topologically protected edge modes in non-equilibrium systems. In other contexts (such as in mechanical and electronic systems) the presence of such edge modes is usually accompanied by a band gap in the spectrum of the system. While the proofs presented here don't make any statements about the band gaps, our numerical results suggest that topologically protected edge modes in non equilibrium systems are indeed accompanied by band gaps in the spectrum of the tilted $W(\lambda)$ matrices. These numerical results are presented in Supplementary Fig. 2. Future work (in preparation) will fully explore connections between localized modes in master equations and localized modes in mechanical and electronic systems in more detail.

## SUPPLEMENTARY DISCUSSION 2: ROBUSTNESS, LOCALIZATION, AND PROTECTION IN FINITE NETWORKS

When the winding number mismatch is $\delta w = 1$ and the probability distribution is localized, the properties of the adaption network described in Fig 4 of the main text can be studied in terms of a model system with a smaller number of states. In the limit of strong localization, we the properties of the adaptive network can be inferred by constructing a simpler four state model as described in Supplementary Fig. 5. The average activity of the four state model is insensitive to changes in the rates along the vertical rungs in the limit that the timescales for transitions along the vertical axis are much smaller than the time scales for transitions along the horizontal axis. In this context

of the original adaptive model these constraints imply that a) $\tau_a \ll \tau_m$, a constraint that is observed in experiments. Since the ligand concentration in the original adaptation model only affects the vertical transition rates in and the rate of transitions along the horizontal methylation axis are ligand concentration and methylation level independent, the effective four state model predicts robust adaptation.

We provide numerical results from adaptation networks with $N = 10$, $N = 20$, and $N = 30$ in Supplementary Fig. 6. Our theoretical predictions are surprisingly accurate even for these values of $N$.


[1] JL Lebowitz and Herbert Spohn, "A Gallavotti–Cohen-type symmetry in the large deviation functional for stochastic dynamics," Journal of Statistical Physics **95**, 333–365 (1999).

[2] Arvind Murugan, David A Huse, and Stanislas Leibler, "Speed, dissipation, and error in kinetic proofreading," Proceedings of the National Academy of Sciences **109**, 12034–12039 (2012).

[3] C. L. Kane and T. C. Lubensky, "Topological boundary modes in isostatic lattices," Nature Physics **10**, 39–45 (2013).
